# Folie à deux: short literature overview and a case report

**DOI:** 10.1192/j.eurpsy.2026.12196

**Published:** 2026-07-31

**Authors:** O. Andrea, B. Zsuzsanna, V. D. W. Ibolya Anna

**Affiliations:** Sántha Kálmán Psychiatric Hospital, Nagykálló, Hungary

## Abstract

**Introduction:**

Treated induced paranoid disorder is rare, therefore, there is insufficient information about the frequency of delusional ideas occurring in family members living with the inducers, as well as about prognosis of the illness. The underlying etiology is primarily attributed to maladaptive family and interpersonal dynamics, as well as psychiatric comorbidities. Four types of shared psychotic disorder were described: imposed, simultaneous, communicated and induced psychosis.

**Objectives:**

We present a case of imposed psychosis in a mother–son dyad, emphasizing clinical features, treatment, and risk factors.

**Methods:**

A 68-year-old woman and her 38-year-old son were admitted with shared somatic delusions and refusal of eating solid food leading to weight loss. They lived in close emotional and physical symbiosis in rural isolation. The mother, a retired seamstress, had a psychiatric history beginning in 2005 with mixed anxiety–depressive disorder, and was later hospitalized for psychotic depression and poor treatment adherence. Brain imaging showed cortical atrophy and microvascular lesions. On current admission, she showed psychomotor retardation, selective mutism, catatonia, and somatic and hypochondriacal delusions. She developed urinary retention and waxy flexibility. Bush–Francis Catatonia Rating Scale indicated marked catatonia (screening 7, severity 24). Neuropsychological testing suggested severe cognitive decline, likely secondary to vascular dementia. She received olanzapine, vortioxetine, and clonazepam. Psychotic symptoms improved significantly (PANSS 6-item: P1 7→2, P2 4→3, P3 1→1, N1 7→5, N4 7→5, N6 7→5). The son had mild lifelong intellectual disability and a history of schizophrenia with multiple hospitalizations and poor adherence. On admission, he was selectively mutistic, withdrawn, and exhibited autistic traits. Treatment with haloperidol and clonazepam led to rapid improvement once separated from his mother; he resumed eating solid food and delusional ideas subsided within days.

**Results:**

After six weeks, both were discharged simultaneously. Socioeconomic hardship necessitated their return to the same environment post-discharge. Local social services were engaged, and institutionalization was advised to minimize relapse risk and ensure supervision.

**Conclusions:**

This case represents a classic form of imposed psychosis, where prolonged cohabitation, emotional dependence, and social isolation enabled the transmission of delusional beliefs from mother to son. Shortly after separation, the induced recovered, and the inducers’ recovery was slower. The inducer’s vascular cognitive impairment and the induced’s pre-existing schizophrenia and intellectual disability likely increased vulnerability. Shared psychotic disorder can emerge in isolated, codependent dyads with pre-existing psychopathology. Early recognition, treatment, separation, and social support are essential for recovery and prevention of recurrence.

**Disclosure of Interest:**

None Declared

